# Optical Encryption Using Attention-Inserted Physics-Driven Single-Pixel Imaging

**DOI:** 10.3390/s24031012

**Published:** 2024-02-04

**Authors:** Wen-Kai Yu, Shuo-Fei Wang, Ke-Qian Shang

**Affiliations:** Center for Quantum Technology Research, Key Laboratory of Advanced Optoelectronic Quantum Architecture and Measurement of Ministry of Education, School of Physics, Beijing Institute of Technology, Beijing 100081, China; 3120195769@bit.edu.cn (S.-F.W.); 3120211559@bit.edu.cn (K.-Q.S.)

**Keywords:** optical encryption, single-pixel imaging, image reconstruction, attention module, physics-driven neural network

## Abstract

Optical encryption based on single-pixel imaging (SPI) has made great advances with the introduction of deep learning. However, the use of deep neural networks usually requires a long training time, and the networks need to be retrained once the target scene changes. With this in mind, we propose an SPI encryption scheme based on an attention-inserted physics-driven neural network. Here, an attention module is used to encrypt the single-pixel measurement value sequences of two images, together with a sequence of cryptographic keys, into a one-dimensional ciphertext signal to complete image encryption. Then, the encrypted signal is fed into a physics-driven neural network for high-fidelity decoding (i.e., decryption). This scheme eliminates the need for pre-training the network and gives more freedom to spatial modulation. Both simulation and experimental results have demonstrated the feasibility and eavesdropping resistance of this scheme. Thus, it will lead SPI-based optical encryption closer to intelligent deep encryption.

## 1. Introduction

Optical encryption mainly uses optical principles and techniques for information encryption and decryption, offering advantages such as high speed, enhanced security, and resistance to jamming and attacks, making it an important development direction in the field of information security. Traditional optical encryption mainly relies on double-random phase-encoding techniques [1,2,3,4], optical interference principles [5,6,7], phase recovery algorithms [8,9,10], and computational holography methods [11,12,13]. However, these methods generally require complex optical systems and sophisticated optical components and are susceptible to environmental noise and optical device errors, resulting in poor encryption and reduced security.

Recently, single-pixel imaging (SPI) has attracted significant attention due to its features such as a simple optical system and low detector cost [14,15,16,17,18]. Unlike classical imaging systems, SPI uses a single-pixel detector instead of a conventional pixelated array detector and recovers the two-dimensional (2D) spatial light-field information of the target by correlating modulation patterns and a one-dimensional (1D) single-pixel (bucket) signal. Its optical modulation and image reconstruction processes can be regarded as the encoding and decoding of the target, respectively, making SPI very suitable for optical image encryption [19,20,21,22,23]. In the image reconstruction process of SPI, the fidelity of decoding is ensured by requiring a sufficiently large number of modulations, whose transmission poses a security risk and also increases the burden of data transmission. We can reduce the number of modulations and improve reconstruction quality by using compressed sensing [24,25], optimized orthogonal Hadamard basis patterns [26,27], or sinusoidal patterns [28,29]. With the advancement of research, deep learning (DL) has also become a mainstream technique adopted in SPI [30,31,32,33,34], which is capable of obtaining high-fidelity images at low sampling rates. To address the problem of poor generalization of the pre-training of data-driven neural networks to target scenes, Situ et al. [35] proposed a physics-driven neural network suitable for SPI. It uses the residuals between experimentally measured single-pixel values and the estimated single-pixel values output by the network to iteratively correct the parameters in the untrained network so that the image reconstruction results constantly approximate the real object images. However, its deep integration with optical encryption needs to be further investigated.

The attention module is currently a popular module structure in neural networks. The introduction of this module allows a network to automatically learn the important features of the input and adjust the weights accordingly [36,37,38]. This mechanism allows a network to focus more on important information, improving the accuracy and robustness of the network. The inputs of this module are three vectors and its output is one vector. Therefore, the forward propagation process of this module can also be regarded as the encryption of these three input vectors. Based on this concept, this paper proposes a physically driven neural network SPI encryption scheme using the above attention module to deal with multi-image encryption tasks [39,40,41,42]. We let the two vectors of the attention module be taken from single-pixel measurements that correspond to two different object images and set the sequence of cryptographic keys as the third vector. The output vector will be transmitted as ciphertext for communication. These three vectors have the same number of elements. In the decryption process, we use a physically driven neural network to decode two sequences of single-pixel measurements from the ciphertext and then reconstruct two decrypted images via repeated iterations of fully connected layers of the deep neural network. The neural network infrastructure used here is the multi-wavelet residual dense convolutional neural network (MWRDCNN) [43], previously proposed by our group, which has been proven to have superior performance. Combined with the physics-driven mechanism, the quality and efficiency of image reconstruction can be further improved. We will verify the feasibility and efficiency of this scheme and its sensitivity to eavesdropping attacks through both numerical simulations and optical experiments. To the best of our knowledge, this scheme is the first attempt at applying the attention module in SPI-based image encryption. Moreover, the use of a physics-driven neural network in the decryption stage makes this method more versatile, gives more design freedoms to optical modulation (without the need to use specifically trained patterns), and decodes higher-quality object images compared to data-driven DL algorithms.

## 2. Principles and Methods

### 2.1. Optical Image Encryption

**Figure 1:**
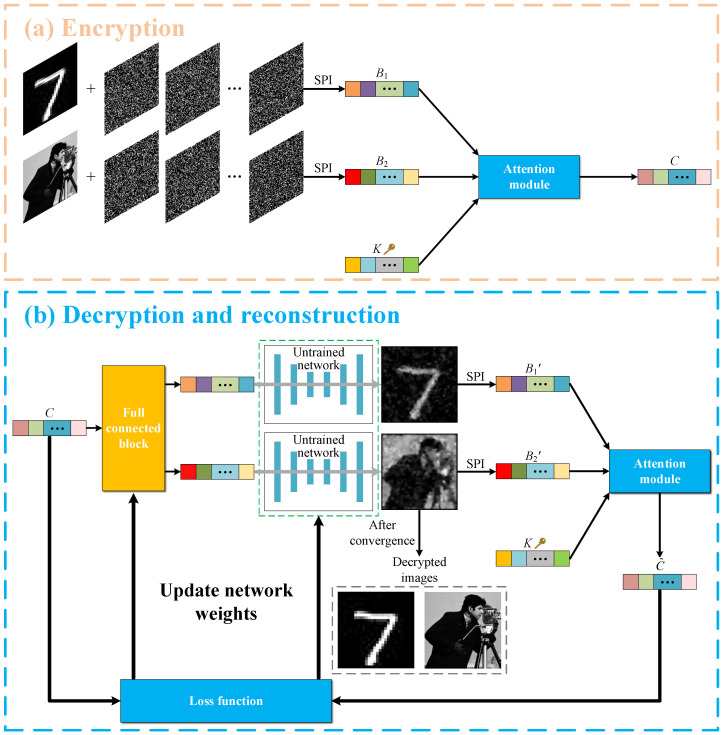
Encryption and decryption processes. (**a**,**b**) Flowcharts of image encryption and decryption and image reconstruction, respectively. SPI denotes the measurement process of single-pixel imaging.

The optical image encryption process in this scheme is shown in Figure 1a. We assume that there are *N* modulation patterns $P_{i}$, each consisting of 0 s and 1 s. These patterns are illuminated onto the object image O(x,y) to be encrypted, and the modulated light field is recorded by a single-pixel (bucket) detector. The measured light intensity of the single-pixel detector $B_{i}$ (i=1,2,⋯,N) can be expressed as:

(1) $$B_{i}=\;\int \int \;P_{i}\left(x,y\right)O\left(x,y\right)dxdy.$$

In this scheme, two object images are encoded using the same number of modulation patterns, yielding two sets of single-pixel measurements, denoted as $B_{1}$ and $B_{2}$, respectively. Then, $B_{1}$ and $B_{2}$ are fed into the attention module along with a sequence of cryptographic keys *K* of the same dimension, which is composed entirely of random real numbers. The output signal of the attention module is the encrypted ciphertext *C*. Since all three inputs to the attention module are purely random, its output is also purely random, and no useful information can be obtained from the encrypted signal *C* alone. The above process can be expressed as:

(2) $$C=\mathrm{Attention}\left(B_{1},B_{2},K\right)=\mathrm{Softmax}\left(\frac{B_{1}\times B_{2}^{T}}{\sqrt{d}}\right)K,$$

where *d* is the number of elements in the sequence of $B_{1}$, $B_{2}$, *K*. After being processed by the attention module, two sequences of single-pixel (bucket) values according to two different object images are encrypted into a ciphertext sequence of the same dimension.

### 2.2. Decryption and Image Reconstruction

In Figure 1b, we illustrate our decryption process. First, the ciphertext sequence passes through a fully connected layer to generate two sequences of 1D signals with the same length as $B_{1}$ and $B_{2}$. These two sequences of signals are then fed into an untrained neural network to complete the initial reconstruction of two object images. Then, we use the SPI mathematical measurement model to compute the single-pixel values, $B_{1}^{\prime }$ and $B_{2}^{\prime }$, of the above initially reconstructed images and input them, together with the sequence of cryptographic keys *K*, into the attention module, which outputs the estimated values $\hat{C}$ of the ciphertext sequence. Next, the residual between the estimated values of the ciphertext $\hat{C}$ and the original ciphertext is calculated to update the weights of the network. With repeated iterations, the residual gradually decreases and the output of the network finally converges to two high-quality plaintext images. The loss (residual) function used here is defined as:

(3) $$L=\frac{1}{2N}\sum_{i=1}^{N}{\left[\hat{C}\left(i\right)-C{\left(i\right)}^{.}\right]}^{2}.$$

### 2.3. Image Reconstruction Neural Network

**Figure 2:**
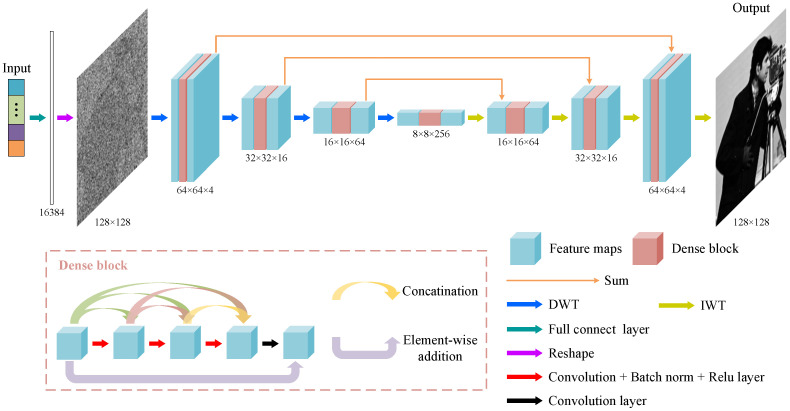
Neural network structure for image reconstruction. DWT and IWT indicate discrete wavelet transform and inverse wavelet transform, respectively.

The applied network architecture for image reconstruction is illustrated in Figure 2. The input 1D signal is augmented by the fully connected layer to a 1 × 16,384 1D signal and reshaped into a 128 × 128 2D image. We continue to use the MWRDCNN proposed in our previous work [43] as the network architecture for image recovery, which has been proven to have superior image reconstruction performance. Compared with the traditional U-net structure, the MWRDCNN replaces the original pooling layer using wavelet and inverse wavelet transforms to complete the upsampling and downsampling processes, respectively, thus avoiding information loss. In each layer of the network, we introduce the dense block that enhances the learning capability of the network by establishing short-term connections among the feature maps. Furthermore, the dense blocks in different layers help the network to accurately extract image features at different scales. After the feature map undergoes the last layer of inverse wavelet transform, the network outputs a reconstructed image, and its weights are optimized based on the calculation of the residuals between the predicted ciphertext and the original ciphertext.

Here, the learning rate decreases exponentially from 0.02 to 0.0002, and an NVIDIA Geforce RTX 2080 Ti GPU is used to accelerate the computation.

## 3. Numerical Simulation and Analysis

**Figure 3:**
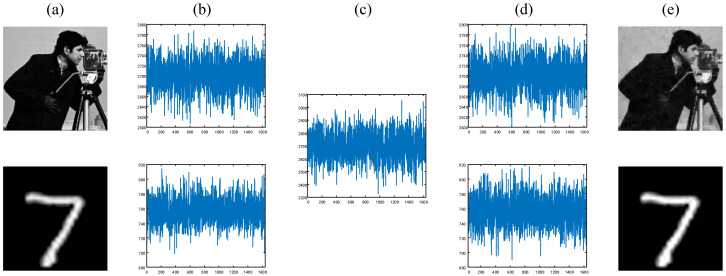
Simulation results with a 10% sampling rate. (**a**) The plaintext image; (**b**) the single-pixel signals; (**c**) the encrypted signal; (**d**) the decrypted single-pixel signal; (**e**) the reconstructed plaintext images.

To verify the feasibility of the proposed approach, some numerical simulations are performed, as shown in Figure 3a. The two images to be encrypted are the “cameraman” image from the “Set12” data set and the handwritten digital image from the MNIST data set. We first resize each image to 128×128 pixels and set the number of modulation patterns to 1638, corresponding to a sampling rate of 10%. The single-pixel intensities simulated from these two images are given in Figure 3b. In Figure 3c, we show the 1D encrypted sequence output by the attention module. With the help of the sequence of cryptographic keys and the physics-driven neural network, two decrypted sequences and two high-quality plaintext images can be acquired, as shown in Figure 3d and Figure 3e, respectively.

### 3.1. Effect of Network Parameters on Reconstruction Results

The MWRDCNN is a typical U-network structure. Generally, deeper network structures often result in slow computation speed and extremely large memory, increasing difficulty in practical applications. Thus, in this paper, we investigate the reconstruction results of a network with different numbers of layers to select an appropriate network structure. It should be noted that the 16 GB of video memory of the used RTX 2080 Ti is not enough to withstand a network with more than four layers. Since the video memory of the RTX 2080 Ti exceeds that of most graphics cards on the market, the network structure with more than four layers is not discussed in this paper.

**Figure 4:**
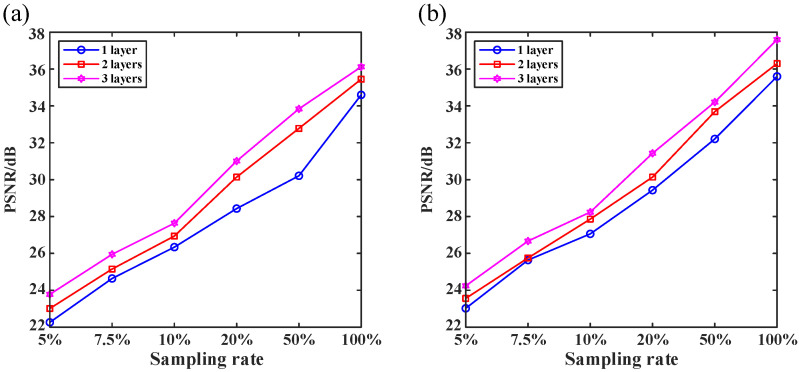
Peak signal-to-noise ratio (PSNR) curves of reconstructed images using our network of different depths. (**a**,**b**) PSNR curves of the “cameraman” and handwritten digit images with different numbers of layers, respectively.

The reconstruction quality using the MWRDCNN with the number of layers changing from one to three at different sampling ratios is presented in Figure 4. We use the peak signal-to-noise ratio (PSNR) as a quantitative measure to evaluate the reconstruction quality:

(4) $$\mathrm{PSNR}=10\log_{10}\left(\frac{1}{\mathrm{MSE}}\right),$$

(5) $$\mathrm{MSE}=\frac{1}{128^{2}}\sum_{p=1}^{128}\sum_{q=1}^{128}{\left[\hat{O}\left(p,q\right)-O\left(p,q\right)\right]}^{2}.$$

where MSE is the mean-square error and $\hat{O}$ denotes the reconstructed image. The larger the PSNR value, the better the reconstruction quality.

It can be observed in Figure 4 that the reconstruction quality of all three networks improves linearly as the sampling rate increases, and deeper networks have better reconstruction. Therefore, in the following, we use the MWRDCNN with a three-layer depth for both the numerical simulations and optical experiments.

### 3.2. Effect of the Number of Training Steps on Reconstruction Quality

**Figure 5:**
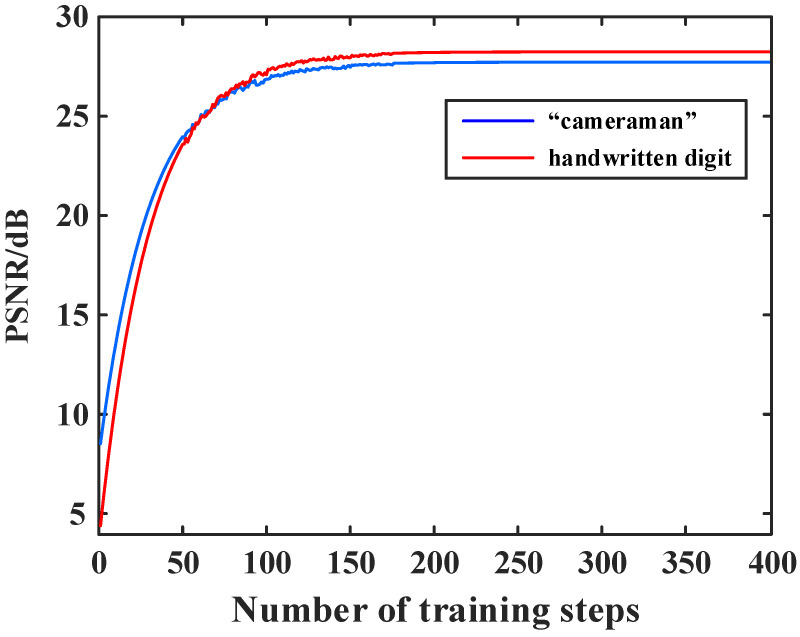
PSNR curves of reconstructed “cameraman” and handwritten digit images as a function of the number of training steps, which is changing from 1 to 400.

To balance the reconstruction quality and computational efficiency, we further investigate the relationship between the reconstruction quality and the number of training steps. The curves of the recovered “cameraman” image and handwritten digit image are plotted in Figure 5.

It can be seen in Figure 5 that when the number of training steps is less than 200, the PSNR value significantly increases with the number of training steps, whereas when the number of training steps is greater than 200, the PSNR value tends to be saturated and no longer significantly increases. Therefore, we set the number of training steps to 200 in the following simulations and experiments.

### 3.3. Effect of the Number of Stolen Bits of the Cryptographic Key Sequence on the Reconstruction Results

**Figure 6:**
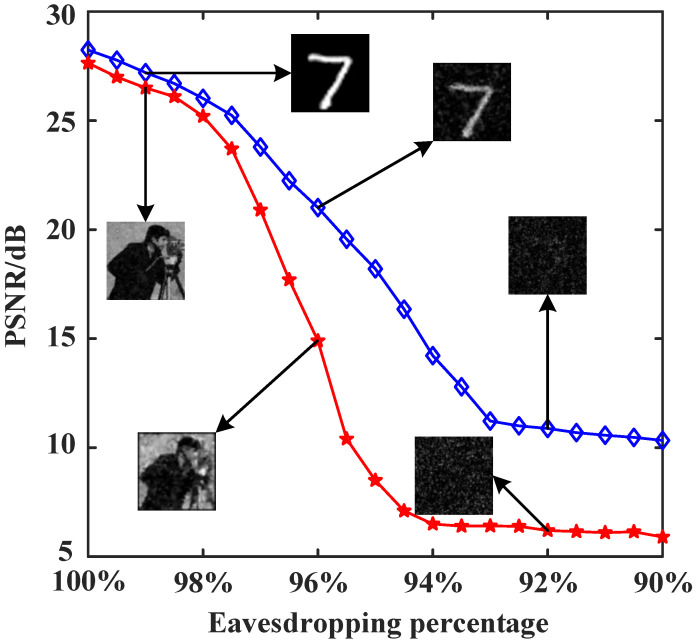
PSNR curves of the “cameraman” and handwritten digit images as a function of the eavesdropping percentage, with a sampling rate of 10%.

To verify the security of the attention-inserted cryptosystem, we perform an eavesdropping analysis. At first, the security of the system is ensured by the privacy of the modulation patterns and cryptographic keys. If the eavesdropper Eve randomly guesses a set of random modulation patterns and cryptographic key sequence, it is absolutely impossible for Eve to decrypt any information. From the perspective of matrix theory, even if the modulation patterns are wrong by just a little bit, the final decryption results will be very different. Now, let us consider an extreme case where Eve has bribed a user to acquire the complete modulation patterns, along with knowledge of the pixel size of the object images, and the cryptographic key with some missing values. Eve does not know whether the intercepted cryptographic keys are complete, and the length of the original cryptographic key sequence is only related to the number of modulations. In the following analysis, we examine the effect of the proportion of correct stolen cryptographic keys to the total number of cryptographic keys on the decryption and reconstruction quality. In Figure 6, we plot the PSNR curves of the “cameraman” and handwritten digit images as a function of the eavesdropping percentage. For the grayscale “cameraman” image, when the eavesdropping ratio is lower than 96%, the reconstructed image does not contain any valuable information. For the handwritten digit image, the reconstruction quality gradually decreases as the eavesdropping ratio decreases, and when the eavesdropping ratio is lower than 92%, no valuable information can be displayed in the recovered image. Thus, very few missing keys can lead to deciphering failures. Furthermore, as long as the length of the cryptographic key sequence is large enough, the proposed cryptosystem will exhibit good resistance to eavesdropping attacks.

## 4. Optical Experiment Results

**Figure 7:**
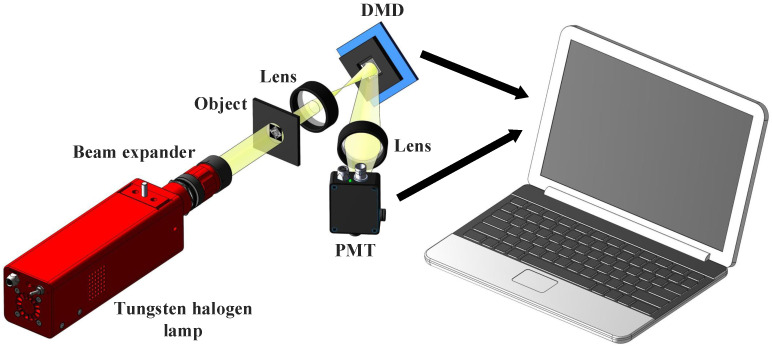
Experimental setup. The thermal light illuminates the object and is then imaged onto a digital micromirror device (DMD), with the modulated total intensity being recorded by a photomultiplier tube (PMT).

We built the SPI’s experimental setup to further verify the proposed method, as shown in Figure 7. The thermal light emitted from a tungsten halogen lamp is collimated and then illuminates the object to be encrypted. The light field that carries the object information is imaged onto a digital micromirror device (DMD) for intensity modulation. The modulated light field is then focused by a lens and collected by a photomultiplier tube (PMT), which outputs a 1D sequence of single-pixel values. In this scheme, the two object images required for a single encryption are encoded in this way.

**Figure 8:**
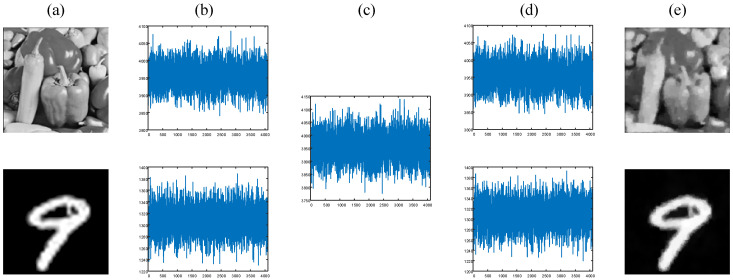
Experimental results with a 25% sampling ratio. (**a**) The grayscale “peppers” image and digit “9” image as the object (plaintext) images; (**b**) the single-pixel measurements of the object images; (**c**) the encrypted signal; (**d**) the decrypted single-pixel signals; (**e**) the recovered plaintext images.

In our experiments, we select the “peppers” image from the “Set12” data set and another handwritten digit image from the MNIST data set as the object (plaintext) images, as shown in Figure 8a. Each object image is modulated 4096 times by the DMD, corresponding to a sampling rate of 25%. Without loss of generality, the switching speed of the DMD is set to 200 frames/s. The recorded single-pixel measurements are presented in Figure 8b. After being processed by the attention module, an encrypted ciphertext sequence can be obtained, as shown in Figure 8c. Then, we use our network to decrypt the single-pixel value sequences and reconstruct the corresponding plaintext images with high quality, as shown in Figure 8d and Figure 8e, respectively. We can see that the performance of this scheme in the optical experiments is consistent with the simulation results, which once again proves the feasibility of this scheme.

**Figure 9:**
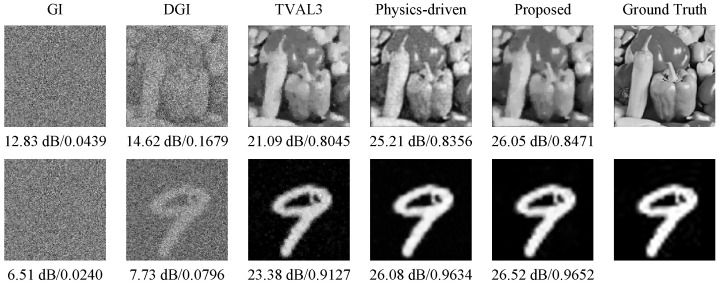
Image reconstruction results (marked with PSNRs and SSIMs) of experimental measurement data with the same sampling rate of 25% using different methods, including background removal GI, differential ghost imaging (DGI), TVAL3, the classical physics-driven neural network, and the proposed method.

Next, to demonstrate the performance of the proposed method in decoding reconstruction, we compare it with a variety of traditional SPI reconstruction algorithms, including background removal GI, differential ghost imaging (DGI), TVAL3, and classical physics-driven neural networks [35], using experimental measurement data. Here, a uniform sampling rate of 25% is chosen, and the reconstruction results are given in Figure 9. To assess the reconstruction quality more comprehensively, we additionally introduce perceptual quantitative metric structural similarity (SSIM) [44], which can evaluate the perceptual difference between the original image and the recovered one. The larger the SSIM value, the better the image quality. Here, we can see that the reconstruction results of traditional intensity correlation functions, such as background removal GI and DGI, are very poor, whereas TVAL3 performs better but still has deficiencies in detail resolution. Both the classical physics-driven neural network and the proposed method are able to reconstruct near-perfect handwritten digital images. But when dealing with the more complicated “peppers” image, more noise exists in the image recovered using the classical physics-driven neural network, and the quality of its decoded image is obviously not as good as the image decoded using our method. Therefore, the proposed method exhibits superior performance in image reconstruction.

**Figure 10:**
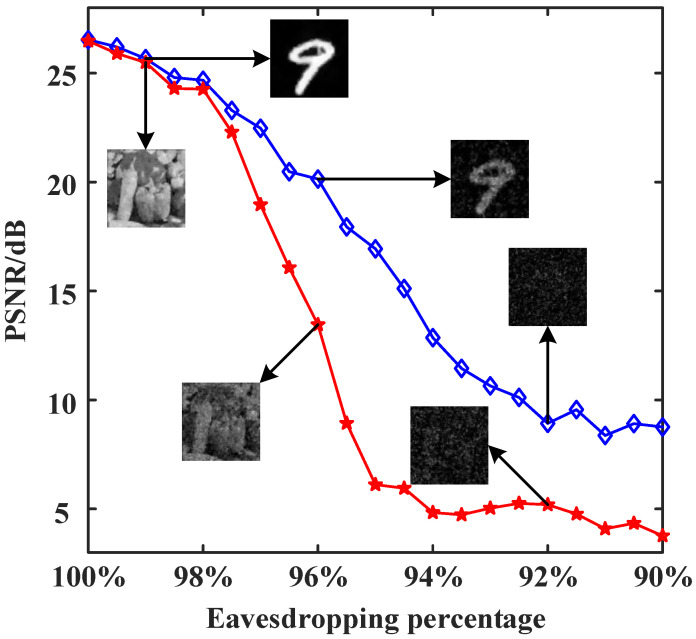
PSNR curves of the reconstructed images from experimental measurements against the eavesdropping percentage of the cryptographic key sequence, with a sampling rate of 25%.

Next, we perform a security analysis using a method similar to that used in the aforementioned simulations. It is also assumed that the eavesdropper can steal some part of the cryptographic key sequence. The PSNR curves of the “peppers” and digit “9” images as a function of the eavesdropping percentage are plotted in Figure 10. The results again prove that the proposed encryption system is sufficiently secure.

In the proposed cryptosystem, the encrypted ciphertext comes from the output of the attention module. The key space [41,42,45] can be infinite because either the two sequences of single-pixel measurements or the input cryptographic key sequence are random with unlimited dynamic range.

**Figure 11:**
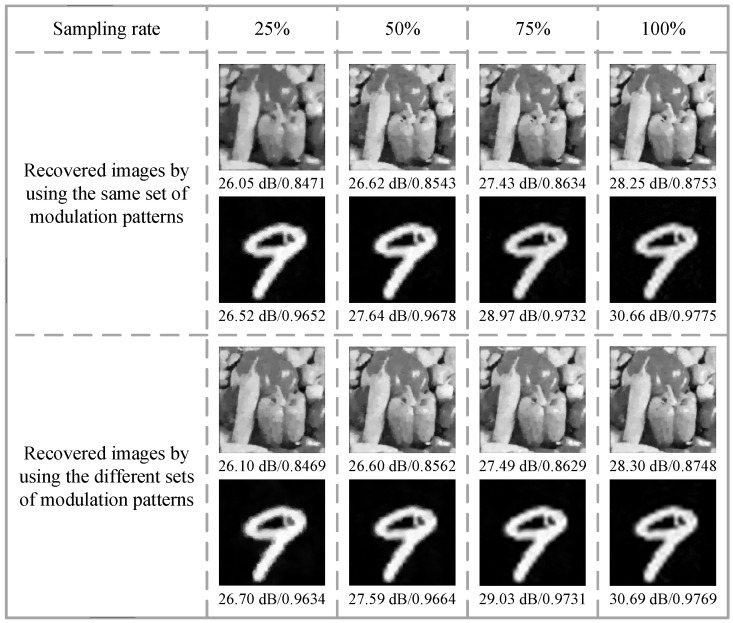
Reconstruction results (marked with PSNRs and SSIMs) for two object images using identical and different sets of modulation patterns.

To prove the generality of the proposed optical encryption scheme, we further analyze the effect of the modulation patterns on the image reconstruction results (see Figure 11).

In the first case, two object images are encoded using two identical sets of modulation patterns, whereas in the second case, they are encoded using two different sets of modulation patterns. It can be seen that in both cases, the grayscale “peppers” image can be reconstructed with high quality at a sampling rate of 25%. The details of the reconstructed images become more enriched with the increase in the sampling rate. The handwritten digit “9” image exhibits excellent recovery when the sampling rate is not less than 25%. Benefiting from the use of the attention module and physics-driven neural network, our encryption method can complete encryption using arbitrary modulation patterns and achieve good decryption results, which are sufficient to meet practical requirements.

## 5. Conclusions

In summary, we propose an efficient optical image encryption method based on attention physics-driven deep SPI. In this method, we use the attention module to perform encryption, with inputs of two sequences of single-pixel values corresponding to two object (plaintext) images and a sequence of cryptographic keys. For the decryption process, we design an attention-inserted physics-driven neural network to constantly update the weights of the network by calculating the residuals between the estimated ciphertext and the true ciphertext, ultimately obtaining high-fidelity reconstruction results. This optimized attention-inserted physics-driven neural network structure not only ensures high-quality reconstruction of plaintext images but can also be easily applied to a wide variety of modulation patterns. The privacy of the modulation patterns and the cryptographic keys is crucial to ensure the security of the system. We have also demonstrated through simulation and experimental results that if the eavesdropper steals some part of the cryptographic keys, deciphering will fail as long as a small number of cryptographic keys are missing. Although the attention module introduced in this paper is only capable of handling two-channel image encryption, in future research, we will design more complicated modules with more input channels to handle multi-image encryption tasks. Therefore, this work tries to break new ground, hoping to provide new ideas for intelligent optical encryption based on SPI.

## Data Availability

Data are contained within the article.

## References

[1] Lin K.T. (2010). Hybrid encoding method for hiding information by assembling double-random phase-encoding technique and binary encoding method. Appl. Opt..

[2] Moon I., Kim Y., Gholami S., Jeong O. (2021). Double random phase encoding schemes with perfect forward secrecy for robust image cryptography. OSA Contin..

[3] Wang Z., Su Y., Wang X., Wang B., Li S., Liu C., Li J., Cai Z., Wan W. (2022). Security-enhanced multiple-image encryption based on quick response codes and modified double random phase encoding in the fractional Fourier transform domain. Appl. Opt..

[4] Tian P., Su R. (2022). A novel virtual optical image encryption scheme created by combining chaotic s-box with double random phase encoding. Sensors.

[5] Zhou L., Xiao Y., Chen W. (2019). Machine-learning attacks on interference-based optical encryption: Experimental demonstration. Opt. Express.

[6] Zhu N., Wang Y., Liu J., Xie J., Zhang H. (2009). Optical image encryption based on interference of polarized light. Opt. Express.

[7] Wang Q., Guo Q., Zhou J. (2013). Multiple-image encryption using polarized light encoding and the optical interference principle in the Fresnel-transform domain. Appl. Opt..

[8] Piao M., Liu Z., Piao Y., Wu H., Yu Z., Kim N. (2018). Multi-depth three-dimensional image encryption based on the phase retrieval algorithm in the Fresnel and fractional Fourier transform domains. Appl. Opt..

[9] Wu J., Wang J., Nie Y., Hu L. (2019). Multiple-image optical encryption based on phase retrieval algorithm and fractional Talbot effect. Opt. Express.

[10] He X., Jiang Z., Kong Y., Wang S., Liu C. (2020). Optical multi-image encryption based on focal length multiplexing and multimode phase retrieval. Appl. Opt..

[11] Muniraj I., Guo C., Malallah R., Ryle J., Healy J., Lee B., Sheridan J. (2017). Low photon count based digital holography for quadratic phase cryptography. Opt. Lett..

[12] Matoba O., Javidi B. (2002). Optical retrieval of encrypted digital holograms for secure real-time display. Opt. Lett..

[13] Kim Y., Sim M., Moon I. (2019). Secure storage and retrieval schemes for multiple encrypted digital holograms with orthogonal phase encoding multiplexing. Opt. Express.

[14] Shapiro J.H. (2008). Computational ghost imaging. Phys. Rev. A.

[15] Katz O., Bromberg Y., Silberberg Y. (2009). Compressive ghost imaging. Appl. Phys. Lett..

[16] He Y., Zhang A., Li M., Huang Y., Quan B., Li D., Wu L., Chen L. (2019). High-resolution sub-sampling incoherent x-ray imaging with a micro-structured scintillator array. Opt. Express.

[17] Xiong J., Zhang Z.-H., Li Z., Zheng P., Li J., Zhang X., Gao Z., Wei Z., Zheng G., Wang S.-P. (2023). Perovskite single-pixel detector for dual-color metasurface imaging recognition in complex environment. Light-Sci. Appl..

[18] Ye Z., Zhou C., Ding C.-X., Zhao J., Jiao S., Wang H.-B., Xiong J. (2023). Ghost diffractive deep neural networks: Optical classifications using light’s second-order coherence. Phys. Rev. Appl..

[19] Liu J., Wang L., Zhao S. (2022). Secret sharing scheme based on spread spectrum ghost imaging. Appl. Opt..

[20] Lin S., Wang X., Zhu A., Xue J., Xu B. (2022). Steganographic optical image encryption based on single-pixel imaging and an untrained neural network. Opt. Express.

[21] Jiao S., Feng J., Gao Y., Lei T., Yuan X. (2020). Visual cryptography in single-pixel imaging. Opt. Express.

[22] Wu J., Li S. (2020). Optical multiple-image compression-encryption via single-pixel Radon transform. Appl. Opt..

[23] Wang X., Lin S., Xue J., Xu B., Chen J. (2023). Information security scheme using deep learning-assisted single-pixel imaging and orthogonal coding. Opt. Express.

[24] Meng S.-Y., Shi W.-W., Ji J., Tao J.-J., Fu Q., Chen X.-H., Wu L.-A. (2020). Super-resolution filtered ghost imaging with compressed sensing. Chin. Phys. B.

[25] Zhou C., Feng D., Wang G., Huang J., Huang H., Liu X., Li X., Feng Y., Sun H., Song L. (2023). Double filter iterative ghost imaging for high quality edge and image acquisition. Opt. Express.

[26] Gong W. (2023). Disturbance-free single-pixel imaging via complementary detection. Opt. Express.

[27] Yu W.-K. (2019). Super sub-Nyquist single-pixel imaging by means of cake-cutting Hadamard basis sort. Sensors.

[28] Hou H.-Y., Zhao Y.-N., Han J.-C., Cao D.-Z., Zhang S.-H., Liu H.-C., Liang B.-L. (2023). Complex-amplitude Fourier single-pixel imaging via coherent structured illumination. Chin. Phys. B.

[29] Deng Z., Qi S., Zhang Z., Zhong J. (2023). Autofocus Fourier single-pixel microscopy. Opt. Lett..

[30] Rizvi S., Cao J., Hao Q. (2020). Deep learning based projector defocus compensation in single-pixel imaging. Opt. Express.

[31] Hu H.-K., Sun S., Lin H.-Z., Jiang L., Liu W.-T. (2020). Denoising ghost imaging under a small sampling rate via deep learning for tracking and imaging moving objects. Opt. Express.

[32] Liu X., Han T., Zhou C., Huang J., Ju M., Xu B., Song L. (2023). Low sampling high quality image reconstruction and segmentation based on array network ghost imaging. Opt. Express.

[33] Peng L., Xie S., Qin T., Cao L., Bian L. (2023). Image-free single-pixel object detection. Opt. Lett..

[34] Peng Y., Chen W. (2023). Learning-based correction with Gaussian constraints for ghost imaging through dynamic scattering media. Opt. Lett..

[35] Wang F., Wang C., Chen M., Gong W., Zhang Y., Han S., Situ G. (2022). Far-field super-resolution ghost imaging with a deep neural network constraint. Light-Sci. Appl..

[36] Wei Z., Wu X., Tong W., Zhang S., Yang X., Tian J., Hui H. (2022). Elimination of stripe artifacts in light sheet fluorescence microscopy using an attention-based residual neural network. Biomed. Opt. Express.

[37] Xi X., Meng X., Qin Z., Nie X., Yin Y., Chen X. (2020). IA-net: Informative attention convolutional neural network for choroidal neovascularization segmentation in OCT images. Biomed. Opt. Express.

[38] Liu H., Zhang Y., Cheng Z., Zhai J., Hu H. (2022). Attention-based neural network for polarimetric image denoising. Opt. Lett..

[39] Li Q., Meng X., Yin Y., Wu H. (2021). A multi-image encryption based on sinusoidal coding frequency multiplexing and deep learning. Sensors.

[40] Perez R.A., Vilardy J.M., Pérez-Cabré E., Millán M.S., Torres C.O. (2023). Nonlinear encryption for multiple images based on a joint transform correlator and the gyrator transform. Sensors.

[41] Feng W., Wang Q., Liu H., Ren Y., Zhang J., Zhang S., Qian K., Wen H. (2023). Exploiting newly designed fractional-order 3D Lorenz chaotic system and 2D discrete polynomial hyper-chaotic map for high-performance multi-image encryption. Fractal Fract..

[42] Qian K., Xiao Y., Wei Y., Liu D., Wang Q., Feng W. (2023). A robust memristor-enhanced polynomial hyper-chaotic map and its multi-channel image encryption application. Micromachines.

[43] Wang S.-F., Yu W.-K., Li Y.-X. (2020). Multi-wavelet residual dense convolutional neural network for image denoising. IEEE Access.

[44] Wang Z., Bovik A.C., Sheikh H.R., Simoncelli E.P. (2004). Image quality assessment: From error visibility to structural similarity. IEEE Trans. Image Process..

[45] Li H., Yu S., Feng W., Chen Y., Zhang J., Qin Z., Zhu Z., Marcin W. (2023). Exploiting dynamic vector-level operations and a 2D-enhanced logistic modular map for efficient chaotic image encryption. Entropy.

